# Number of chronic conditions and associated functional limitations among older adults: cross-sectional findings from the longitudinal aging study in India

**DOI:** 10.1186/s12877-021-02620-0

**Published:** 2021-11-23

**Authors:** Palak Sharma, Priya Maurya, T. Muhammad

**Affiliations:** 1 Department of Mathematical Demography and Statistics, International Institute for Population Sciences, Mumbai, Maharashtra 400088 India; 2 Department of Development Studies, International Institute for Population Sciences, Mumbai, Maharashtra 400088 India; 3 Department of Population Policies and Programmes, International Institute for Population Sciences, Deonar, Mumbai, Maharashtra 400088 India

**Keywords:** Chronic conditions, ADL and IADL, Older adults, LASI

## Abstract

**Background:**

Chronic conditions reduce the likelihood of physical functioning among older adults. However, the contribution of most prevalent diseases and multimorbidity to different measures of functional limitations is relatively underexplored among Indian older adults. The present study explores the prospective association between number of chronic conditions and limitations in activities of daily living (ADL) and instrumental activities of daily living (IADL) among older adults in India.

**Methods:**

This study utilized data from the nationally representative Longitudinal Ageing Study in India (LASI-2017-18). The effective sample size was 31,464 older adults aged 60 years and above. Descriptive statistics along with cross-tabulation were presented in the study. Additionally, binary logistic regression analysis was used to fulfil the objectives. The outcome variables were dichotomized; high representing no difficulty in ADL/IADL and low representing a difficulty in at least one ADL/IADL. The chronic conditions included hypertension, diabetes, neurological/psychiatric disease, lung disease, heart diseases, stroke, and bone-related disease. The number of chronic diseases was categorized into no disease, single, two and three plus based on number of reported disease.

**Results:**

26.36% of older women and 20.87% of older men had low ADL and the figures for low IADL were 56.86 and 38.84% for older men and women respectively. The likelihood of low ADL (AOR: 1.698, CI:1.544, 1.868) and low IADL (AOR: 1.197; CI: 1.064, 1.346) was higher among womenthan men. With increasing age, the prevalence of low ADL increased among older adults. Respondents with pre-existing chronic conditions had higher likelihood of low ADL and IADL. Older adults with hypertension, psychiatric disease, heart disease, stroke and bone-related disease had significantly higher odds of reporting low IADL. The chances of low ADL and IADL were 2.156 (CI: 1.709, 2.719) and 2.892 (CI: 2.067, 4.047) times respectively higher among older adults with more than three chronic conditions. After controlling for socio-economic and health-related covariates, it was found that men with more than three pre-existing chronic conditions had higher odds of low ADL than women. On the other hand, low IADL were found higher among women with more than three pre-existing chronic conditions.

**Conclusions:**

The present study demonstrates a significant burden of functional limitations among older individuals and that there is a strong association between pre-existing chronic conditions and functional disability. Those with hypertension, diabetes, psychiatric disorders, heart disease, stroke, lung disease or bone-related diseases should be effectively monitored to predict future functional limitations, which may lead to worsening health.

**Supplementary Information:**

The online version contains supplementary material available at 10.1186/s12877-021-02620-0.

## Background

Worldwide, the population is experiencing a phase of rapid aging, accompanied by remarkable changes in disease profile where chronic disease with multiple conditions in advanced age is taking central place [1, 2]. However, population ageing is more challenging in middle-income countries due to witnessing an unprecedented upward shift in life expectancy by these countries [3]. According to the latest census of India (2011), the 60 plus population accounted for 8.6% of India’s total population [4]. It is expected to increase to 19.1% of the total population of India by 2050 [3]. This cusp of age structure poses an important public health implication as do the economic and social costs on healthcare [5].

Evidence from both developed and developing countries found a positive association between multimorbidity and age [6–8]. Multimorbidity is commonly defined as the coexistence of two or more chronic conditions requiring long-term care [9]. It leads to adverse health outcomes such as disability, poor self-rated health, reduced quality of life, hospitalization and mortality [1, 8, 10, 11]. The International Classification of Functioning, Disability and Health defines disability as a multidimensional condition of people for impairment, activity limitation and participation restrictions to interact around the world [12]. A person’s ability to perform activities of daily living (ADL) and instrumental ADL is commonly used in epidemiological and clinical studies to assess physical functioning [10]. In the year 2017, around 51% of the years of life lost and lived with disability was attributed to age-related diseases [7] and 23% of the total burden of diseases occurred among the elderly [8]. According to the 2011 census of India, any disability prevalence was found to be 20.82% in the elderly at the national level [13].

The relationship between physical disability and chronic disease and combined effects of multimorbidity have also been explored previously [11, 14, 15]. Chronic multimorbidities reduce the likelihood of physical functioning among older adults [15]. The most prevalent diseases associated with functional limitation are arthritis, diabetes, cardiovascular disease, osteoporosis, lung disorders and hypertension [14, 16]. Klijis et al. found that musculoskeletal diseases (arthritis and back pain) and cardiovascular diseases was associated with ADL/IADL disability among elderly [14]. Likewise, stroke, hypertension, diabetes, arthritis and stroke were the most prevalent chronic condition for disability among Brazilian older adults [16]. Raina et al. [17] reported gender differences in association between chronic diseases and functional limitations. The cardiometabolic domain was the most attributed among men, whereas the musculoskeletal domain was common among women [17]. The recent estimates of contribution of chronic disease to functional disability in India (2011–2012) showed that hypertension, diabetes and cardiovascular disease were the most attributed conditions among population aged 60 and above [15]. Indian older adults with more than two chronic diseases were six times higher in the risk of performing ADL [18]. Similarly, the likelihood of functional limitation was five times higher among Chinese older individuals having more than four chronic conditions [19].

Biological, psychosocial and behavioural factors, including low socio-economic status, poor nutritional intake, lack of education, direct and indirect influence of adverse events of life, health behaviour and poor access to health services, may have complex inter-linkage between multimorbidity and functional limitations, especially among older adults [6, 10, 19]. The contribution of prevalence of multimorbidity to burden of functional limitation was higher in advanced age [10, 14]. Previous studies reported that women were more susceptible to chronic conditions and disability [15, 16, 19]. Older people who belong to the poorest wealth quintile and have a low level of education were at greater risk of vulnerability to the disability, which might be due to lack of knowledge and access to public health services [6, 15].

The rising burden of comorbidities with functional limitations with greater healthcare costs results in economic vulnerability and the declining physical and mental well-being of older adults. However, the contribution of most prevalent diseases and multimorbidity to different measures of functional limitation is relatively under-explored among Indian older adults due to lack of nationally representative data. Hence, the present study explores the prospect association between number of chronic conditions and functional limitation among older adults in India. This study also examines whether the impact of number of chronic conditions on functional limitations varies by socio-demographic characteristics. The present study may be valuables for policymakers to orient health services for target-specific groups, especially among older adults, considering they are at the highest risk of chronic diseases and most vulnerable group for functional disability.

## Methods

### Study design and sample

A cross-sectional study design was adopted. The study utilizes data from India’s first nationally representative Longitudinal Ageing Study (LASI- wave 1) conducted during 2017–18, which investigates the health, economic and social determinants and consequences of population ageing in India [20]. The sample in the LASI survey included 72,250 individuals aged 45 and above and their spouses across all states and union territories of India except Sikkim. Households with at least one member aged 45 and above were taken as the eventual unit of observation. This study provides scientific evidence on demographics, household economic status, chronic health conditions, symptoms-based health condition, functional and mental health, biomarkers, health care utilization, work and employment etc. It enables the cross-state analyses and the cross-national analyses of ageing, health, economic status and social behaviours and has been designed to evaluate the effect of changing policies and behavioural outcomes in India. Detailed information on the sampling frame is available on the LASI wave-1 Report [20]. The effective sample size for the present study was 31,464 older adults.

### Procedure

The survey adopted a three-stage sampling design in rural areas and a four-stage sampling design in urban areas. In each state/UT, the first stage involved the selection of Primary Sampling Units (PSUs), that is, sub-districts (Tehsils/Talukas), and the second stage involved the selection of villages in rural areas and wards in urban areas in the selected PSUs. In rural areas, households were selected from selected villages in the third stage; sampling in urban areas involved an additional stage. Specifically, in the third stage, one Census Enumeration Block (CEB) was randomly selected in each urban area. In the fourth stage, households were selected from this CEB. Further, an individual survey schedule was administered to each consenting respondent aged 45 and above and their spouse (irrespective of age) in the sampled households. The response rate was 95.6% for the individual interviews and 92.7% in household interviews.

### Outcome variables

The outcome variables were dichotomized i.e., ADL (Activities of Daily Living) and IADL (Instrumental Activities of Daily Living) were coded as high and low; high representing no difficulty in ADL/IADL and low representing a difficulty in at least one ADL/IADL.

1. ADL is a term used to refer to normal daily self-care activities (such as movement in bed, changing position from sitting to standing, feeding, bathing, dressing, grooming, personal hygiene etc.) The ability or inability to perform ADLs is used to measure a person’s functional status, especially in the case of people with disabilities and the older adults [21, 22].
2. IADL functions are those which are not necessarily related to fundamental functioning of a person, but they let an individual live independently in a community. The set ask were necessary for independent functioning in the community. Respondents were asked if they were having any difficulties that were expected to last more than 3 months, such as preparing a hot meal, shopping for groceries, making a telephone call, taking medications, doing work around the house or garden, managing money (such as paying bills and keeping track of expenses), and getting around or finding an address in unfamiliar places [21, 22].

### Key explanatory variable

The main explanatory variables were a number of chronic conditions. The diseases were self-reported as was assessed through the question “Has any health professional ever diagnosed you with the following chronic conditions or diseases?”. Chronic conditions included hypertension, diabetes, neurological/psychiatric disease, lung disease, heart diseases, stroke, and bone-related disease [20]. Further, the number of chronic diseases was categorized into no disease, single, two and three plus based on number of reported disease [23].

### Others explanatory variables

#### Individual factors

i. Age was categorized into groups of 60–69 years, 70–79 years and 80+ years.
ii. Sex was categorized as male and female.
iii. Educational status was categorized as no education/primary, secondary and higher.
iv. Work status was recoded into never worked, not working, working and retired.
v. Living arrangement was categorized as living alone, with spouse, and others.
vi. Marital status was coded as currently in union and not in union [24]. Not in union included respondents who were widowed/separated/divorced/never married.

#### Health factors

i. Self-rated health (SRH) was coded as good which includes very good, good and fair whereas, poor includes poor and very poor [25].
ii. Major depression among the older adults was calculated using the CIDI-SF (Short Form Composite International Diagnostic Interview) with a score of 3 or more being coded as 1 for “diagnosed with depression” and less than 3 coded as 0 for “not diagnosed with depression”. This scale estimates a probable psychiatric diagnosis of major depression and has been validated in field settings and widely used in population-based health surveys [26]. The lowest 10th percentile is used as a proxy measure of severe depression among older adults [20].
iii. Cognitive impairment was measured through five broad domains (memory, orientation, arithmetic function, executive function, and object naming). These cognitive measures were derived from the cognition module of Health and Retirement Study (HRS) [27]. Memory was measured using immediate word recall, delayed word recall; orientation was measured using time and place measure, arithmetic function was measured through backward counting, serial seven, and computation method; executive function was measured through paper folding and pentagon drawing method, and object naming was lastly done to measure the cognitive impairment among older adults. A composite score of 0–43 was computed using the multiple domain-wise measures. The lowest 10th percentile is used as a proxy measure of poor cognitive functioning [28].

#### Household factors

i. The monthly per capita expenditure (MPCE) quintile was assessed using household consumption data. Sets of 11 and 29 questions on the expenditures on food and non-food items, respectively, were used to canvas the sample households. Food expenditure was collected based on a reference period of 7 days, and non-food expenditure was collected based on reference periods of 30 days and 365 days. Food and non-food expenditures have been standardized to the 30-day reference period. The monthly per capita consumption expenditure (MPCE) is computed and used as the summary measure of consumption [20]. The variable was then divided into five quintiles i.e., from poorest to richest.
ii. Religion was coded as Hindu, Muslim, and Others.
iii. Caste was recoded as Scheduled Caste/Scheduled Tribe (SC/ST), Other Backward Class (OBC), and others. The Scheduled Caste include “untouchables”; a group of the population that is socially segregated and financially/economically by their low status as per Hindu caste hierarchy. The SCs and STs are among the most disadvantaged socio-economic groups in India. The OBC is the group of people who were identified as “educationally, economically and socially backward”. The OBCs are considered low in the traditional caste hierarchy but are not considered untouchables. The “other” caste category is identified as having higher social status [29].
iv. Place of residence was categorized as rural and urban.

### Statistical analysis

Univariate analyses and cross-tabulations were conducted in the study. Additionally, binary logistic regression analysis [30] was used to establish the association between the outcome variables (low ADL and IADL) and other explanatory variables.

The binary logistic regression model is usually put into a more compact form as follows:

$$\mathrm{Logit}\ \left[\mathrm{P}\left(\mathrm{Y}=1\right)\right]={\beta}_0+\beta \ast X$$

The parameter *β*_0_ estimates the log odds of functional impairments (ADL and IADL) for the reference group, while *β* estimates the maximum likelihood, the differential log odds of functional impairment associated with a set of predictors X, as compared to the reference group.

For a better understanding of the association of number of chronic conditions with functional impairments, we categorized number of chronic conditions into single, two and three plus, and regressed on low ADL and IADL stratified by sex of the older adults.

## Results

Table 1 shows the socio-economic and health profile of the older adults from the study population. 58.51% of the sample ages 60–69 years, 74.02% were illiterate or had primary level formal education, 82.20% follow Hindu Religion, and 70.55% reside in rural parts of India. 42.02% of the men were currently working whereas only 18.87% of women were engaged in paid work. 26.36% of females and 20.87% of males had low ADL, and the figures for low IADL were 38.84 and 56.86% for older male and female, respectively. The prevalence of hypertension (37.10%) and bone-joint-related diseases (22.79%) was higher among females. On the other hand, lung diseases, heart diseases, and strokes were higher among males than females.

**Table 1:** Socio-economic and health profile of the study participants

Background Factors	Total (***N*** = 31,464)		Male (***N*** = 14,931)		Female (***N*** = 16,533)	
	N	%	N	%	N	%
**Hypertension**^**a**^						
No	21,095	67.22	10,836	72.05	10,279	62.9
Yes	10,287	32.78	4205	27.95	6062	37.1
**Diabetes**^**a**^						
No	26,908	85.74	12,841	85.39	14,065	86.06
Yes	4473	14.26	2197	14.61	2278	13.94
**Psychiatric disease**^**a**^						
No	30,500	97.19	14,632	97.3	15,869	97.1
Yes	880	2.81	407	2.70	473	2.90
**Lung disease**^**a**^						
No	28,723	91.52	13,686	90.99	15,035	92
Yes	2660	8.48	1355	9.01	1307	8.00
**Heart disease**^**a**^						
No	29,754	94.81	14,167	94.19	15,584	95.36
Yes	1629	5.19	874	5.81	758	4.64
**Stroke**^**a**^						
No	30,524	97.27	14,246	96.71	15,976	97.77
Yes	858	2.73	495	3.29	365	2.23
**Bone-related disease**^**a**^						
No	25,199	80.29	12,596	83.75	12,618	77.21
Yes	6185	19.71	2445	16.25	3725	22.79
**ADL**^**a**^						
High	23,887	76.23	11,881	79.13	12,019	73.64
Low	7449	23.77	3133	20.87	4303	26.36
**IADL**^**a**^						
High	16,162	51.64	9175	61.16	7029	43.14
Low	15,133	48.36	5827	38.84	9264	56.86
**Age (in years)**						
60–69	18,410	58.51	8730	57.82	9678	59.13
70–79	9501	30.20	4702	31.14	4803	29.35
80+	3553	11.29	1666	11.04	1886	11.52
**Educational status**						
No/primary	23,289	74.02	9202	60.95	14,046	85.82
Secondary	5741	18.24	3958	26.22	1808	11.04
Higher	2434	7.74	1937	12.83	513	3.13
**Work status**						
Never worked	8315	26.43	578	3.83	7665	46.84
Currently not working	11,470	36.45	6173	40.88	5311	32.45
Working	9397	29.87	6348	42.05	3088	18.87
Retired	2282	7.25	1999	13.24	302	1.84
**Marital status**						
Currently in union	19,391	61.63	12,242	81.09	7211	44.06
Not in union	12,072	38.37	2856	18.91	9155	55.94
**MPCE quintile**						
Poorest	6829	21.70	3145	20.83	3681	22.49
Poorer	6831	21.71	3219	21.32	3611	22.06
Middle	6590	20.95	3262	21.60	3331	20.35
Richer	6038	19.19	2902	19.22	3136	19.16
Richest	5175	16.45	2570	17.02	2607	15.93
**Religion**						
Hindu	25,871	82.20	12,386	82.04	13,484	82.39
Muslim	3548	11.30	1769	11.72	1781	10.88
Others	2045	6.50	943	6.25	1101	6.73
**Caste**						
SC/ST	8505	27.10	4001	26.50	4501	27.50
OBC	14,231	45.20	6925	45.86	7308	44.66
Others	8729	27.70	4172	27.63	4556	27.84
**Place of residence**						
Urban	22,196	29.45	4219	27.95	5044	30.82
Rural	9268	70.55	10,879	72.05	11,322	69.18

*SRH* Self-Rated Health, *ADL* Activities of daily living, *IADL* Instrumental activities of daily living, *MPCE* Monthly per capita consumption expenditure

^a^Sample size may differ due to missing cases

Table 2 shows the results from bivariate and logistic regression estimates for low ADL and IADL disabilities in older adults with different chronic conditions and socio-economic backgrounds. Socio-economic factors like age, gender, education, work status and place of residence were common factors that affect the low ADL and IADL in older adults of India. Females were more vulnerable in terms of low ADL and IADL scores. The likelihood of low ADL (AOR: 1.698, CI:1.544, 1.868) and low IADL (AOR: 1.197; CI: 1.064, 1.346) was higher among females than males. With the increase in age, the proportion with low ADL score (higher ADL disability) increases, whereas the proportion decreased in IADL limitation. With the increase in educational attainment, the odds of low ADL score decreases (secondary education: 0.571, higher education: 0.408), and respondents with higher education have 30% fewer chances of reporting low IADL score. Older adults who previously worked but currently not working were 28% more likely to have low ADL (AOR: 1.289, CI: 1.126, 1.499) and low IADL scores (AOR: 1.285, CI: 1.136, 1.476). Those who were not in a marital union have 34% higher chances of reporting low ADL scores. Respondents belonging to the richer wealth quintile are 20% less likely to report low IADL as compared to those in the poorest quintile. Rural residents had a higher proportion of older adults with low ADL (24.42%) and low IADL (51.57%) as compared to urban residents. The respondents residing in rural areas were 55% (AOR:1.557, CI:1.387, 1.748) more likely to have low ADL and had a 12% (AOR:1.127, CI: 0.978, 1.30) higher likelihood of having low IADL score as compared to their urban counterparts.

**Table 2:** Bivariate and logistic regression estimates for ADL and IADL limitations by chronic conditions and other background characteristics

Variables	Low ADL		Low IADL		Low ADL	Low IADL
	%	*p* < 0.05	%	*p* < 0.05	AOR (95% CI)	AOR (95% CI)
**Sex**		0.001		0.001		
Male	20.87		38.84		Ref.	Ref.
Female	26.36		56.86		1.698*** (1.544–1.868)	1.197*** (1.064–1.346)
**Hypertension**		0.001		0.001		
No	22.13		46.52		Ref.	Ref.
Yes	27.14		52.1		1.112** (1.006–1.227)	1.156** (1.030–1.297)
**Diabetes**		0.001		0.662		
No	23.9		48.13		Ref.	Ref.
Yes	23.02		49.75		1.263*** (1.083–1.472)	0.920 (0.777–1.090)
**Psychiatric disease**		0.001		0.001		
No	23.02		47.62		Ref.	Ref.
Yes	49.73		73.51		2.450*** (1.951–3.071)	2.455*** (1.966–3.060)
**Lung disease**		0.001		0.001		
No	23.38		47.18		Ref.	Ref.
Yes	28.02		60.98		1.504*** (1.286–1.759)	1.004 (0.802–1.234)
**Heart disease**		0.001		0.001		
No	23.36		47.91		Ref.	Ref.
Yes	31.47		56.4		1.430*** (1.130–1.810)	1.383* (1.002–1.910)
**Stroke**		0.001		0.001		
No	22.99		47.62		Ref.	Ref.
Yes	51.83		74.3		3.347*** (2.594–4.319)	3.281*** (2.610–4.122)
**Bone-related disease**		0.001		0.001		
No	20.52		44.74		Ref.	Ref.
Yes	37.04		63.07		1.931*** (1.690–2.204)	2.158*** (1.907–2.443)
**Age (in years)**		0.001		0.001		
60–69	17.73		59.15		Ref.	Ref.
70–79	27.94		44.92		1.623*** (1.468–1.794)	1.681*** (1.487–1.901)
80+	44.08		30.53		2.909*** (2.378–3.558)	3.423*** (2.871–4.082)
**Educational status**		0.001		0.001		
No/primary education	25.99		54.17		Ref.	Ref.
Secondary	17.72		34.86		0.571*** (0.494–0.660)	0.690*** (0.577–0.826)
Higher	16.46		23.8		0.408*** (0.328–0.507)	0.701*** (0.537–0.914)
**Work status**		0.001		0.001		
Never worked	25.72		54.31		Ref.	Ref.
Currently not working	30.81		57.61		1.289*** (1.126–1.499)	1.285*** (1.136–1.476)
Working	13.59		35.63		0.651*** (0.566–0.762)	0.760*** (0.677–0.877)
Retired	23.55		33.02		1.388** (1.098–1.779)	1.018 (0.827–1.278)
**Marital status**		0.001		0.001		
Currently in union	20.75		41.47		Ref.	Ref.
Not in union	28.59		59.34		1.341*** (1.190–1.515)	1.070 (0.926–1.235)
**Living arrangement**		0.001		0.001		
Alone	27.27		59.5		Ref.	Ref.
With spouse	21.52		43.58		1.049 (0.846–1.302)	1.060 (0.834–1.348)
With others	24.1		48.77		1.019 (0.845–1.229)	1.015 (0.831–1.240)
**MPCE quintile**		0.187		0.001		
Poorest	25.59		50.35		Ref.	Ref.
Poorer	23.78		49.68		0.977 (0.864–1.103)	0.885* (0.770–1.018)
Middle	23.82		45.89		0.852** (0.746–0.972)	0.890 (0.766–1.035)
Richer	22.4		48.68		0.961 (0.821–1.120)	0.804** (0.680–0.951)
Richest	22.9		46.68		0.916 (0.793–1.057)	0.823* (0.675–1.004)
**Religion**		0.001		0.001		
Hindu	23.49		48.33		Ref.	Ref.
Muslim	26.2		48.98		0.956 (0.823–1.111)	1.066 (0.921–1.235)
Others	23.27		47.6		0.979 (0.845–1.134)	0.981 (0.820–1.175)
**Caste**		0.001		0.001		
SC/ST	23.85		49.02		Ref.	Ref.
OBC	23.03		50.53		1.160*** (1.045–1.288)	0.954 (0.841–1.082)
Others	24.9		44.2		0.989 (0.876–1.115)	1.096 (0.957–1.256)
**Place of residence**		0.313		0.001		
Urban	22.2		40.53		Ref.	Ref.
Rural	24.42		51.57		1.557*** (1.387–1.748)	1.127* (0.978–1.300)
**Total**	23.77		48.36			

*%* Percentage, *Ref* Reference, *AOR* Adjusted Odds Ratio, *ADL* Activities of daily living, *IADL* Instrumental activities of daily living, *MPCE* Monthly per capita consumption expenditure

*if *p* < 0.05, **if *p* < 0.01, ***if *p* < 0.001

Overall, respondents with pre-existing chronic conditions have a higher likelihood of low ADL and IADL scores (hence higher disability in ADL and IADL). The prevalence of low ADL score and low IADL score was higher among those suffering from chronic conditions like hypertension (ADL: 27.14%, IADL: 52.1%), psychiatric disease (ADL: 49.73%, IADL: 73.51%), lung disease (ADL: 28.02, 60.98%), heart disease (ADL: 31.47, 56.4%), stroke (ADL: 51.83%, IADL: 74.3%), and bone-related diseases (ADL: 37.04%, IADL: 63.07%). Respondents with psychiatric disorders and strokes have 1.452 and 2.347 times higher odds of low levels of ADL, respectively. Older adults with hypertension, psychiatric disease, heart disease, stroke, and bone-related disease have significantly higher odds of reporting low IADL scores (OR hypertension: 1.156, psychiatric disease: 2.458, heart disease: 1.383, strokes: 3.283, bone-related: 2.158). Age, education, marital status and place of residence were significantly associated with low ADL and low IADL.

Table S1 (supplementary) shows the results from the logistic regression analysis for the number of chronic conditions and the low ADL and IADL. Results show that the odds of low ADL and IADL increased significantly with an increase in the number of pre-existing chronic conditions among older adults. The chances of low ADL and low IADL were 2.156 (CI: 1.709, 2.719) and 2.892 (CI: 2.067, 4.047) higher among older adults with more than three chronic conditions after adjusting for background characteristics (Table S1).

Table 3 shows sex-specific results of the logistic regression of ADL and IADL disabilities with a number of pre-existing chronic conditions in older adults adjusting for different individual-level, household-level, and health-related indicators under three different models. Overall, males have higher odds of low ADL as compared to females aged 60 years and above. With the increase in the number of chronic conditions, the likelihood of low ADL and low IADL increases under all three models. After adjusting for individual factors (model 1), males with more than three chronic conditions have higher odds of ADL disability as compared to their female counterparts (OR males: 3.575, females: 2.470). Additionally, when controlled for household level factors, the odds of low ADL decrease for males but slightly increase for females. Male respondents with two chronic conditions are 88% more likely to have low ADL, whereas females are two times more likely to have a low ADL score in comparison to those who have no pre-existing chronic condition. In the case of IADL disability, females have overall higher odds of low IADL scores as compared to males with the increase in the number of chronic conditions. After adjusting for individual-level factors in model 1, females with three or more chronic conditions show 3.39 times higher odds of low IADL score, whereas, for males, the odds are as high as 3 times. Under model 3, with the increase in the number of chronic conditions in males from one, two, and three-plus diseases, the likelihood of low IADL increases from 31 to 128%. On the other hand, females with three or more chronic conditions have 3.31 times higher odds of low IADL scores (higher disability) when controlled for all possible covariates in the model.

**Table 3:** Sex stratified logistic regression estimates of low ADL and low IADL by number of chronic conditions

Number of chronic diseases	Unadjusted	AOR (95% CI)	AOR (95% CI)	AOR (95% CI)
		Model 1	Model 2	Model 3
**Low ADL**				
Male				
No disease	Ref.	Ref.	Ref.	Ref.
Single disease	1.599*** (1.328–1.927)	1.618*** (1.349–1.941)	1.540*** (1.283–1.849)	1.474*** (1.191–1.825)
Two	1.921*** (1.556–2.371)	2.058*** (1.672–2.532)	1.886*** (1.523–2.336)	1.666*** (1.285–2.160)
Three and above	3.067*** (2.451–3.838)	3.575*** (2.833–4.511)	3.203*** (2.523–4.068)	2.499*** (1.889–3.304)
Female				
No disease	Ref.	Ref.	Ref.	Ref.
Single disease	1.340*** (1.140–1.576)	1.329*** (1.124–1.570)	1.332*** (1.128–1.573)	1.174* (0.980–1.405)
Two	1.979*** (1.677–2.336)	2.077*** (1.755–2.459)	2.094*** (1.744–2.515)	1.793*** (1.457–2.206)
Three and above	2.162*** (1.440–3.247)	2.470*** (1.825–3.343)	2.591*** (1.950–3.444)	1.955*** (1.405–2.719)
**Low IADL**				
Male				
No disease	Ref.	Ref.	Ref.	Ref.
Single disease	1.360*** (1.187–1.557)	1.439*** (1.252–1.655)	1.414*** (1.224–1.633)	1.310*** (1.111–1.545)
Two	1.538*** (1.289–1.835)	1.835*** (1.548–2.175)	1.760*** (1.488–2.083)	1.502*** (1.236–1.826)
Three and above	2.231*** (1.836–2.709)	3.009*** (2.442–3.708)	2.934*** (2.358–3.649)	2.285*** (1.796–2.908)
Female				
No disease	Ref.	Ref.	Ref.	Ref.
Single disease	1.265*** (1.106–1.447)	1.284*** (1.111–1.484)	1.343*** (1.167–1.546)	1.316*** (1.134–1.527)
Two	1.511*** (1.299–1.756)	1.642*** (1.416–1.905)	1.776*** (1.520–2.076)	1.511*** (1.276–1.788)
Three and above	2.671*** (1.875–3.805)	3.396*** (2.292–5.030)	3.602*** (2.467–5.258)	3.310*** (2.044–5.362)

Model 1 is adjusted for age, education, work status, marital status and living arrangement; Model 2 is adjusted for individual as well as household factors (wealth quintile, religion, caste, place of residence); Model 3 is additionally adjusted for SRH, depression and cognitive impairment

## Discussion

The presence of functional disability among older adults has become an important public health problem. Many previous studies have established its association with multiple chronic conditions in individuals, but it has been underexplored in the Indian setting. The present study focused on the association of multiple pre-existing chronic conditions with the functional limitations (ADL and IADL) in older adults of India and how it varies for population in different socioeconomic and demographic strata.

According to the current results, in India, around 24 and 48% of older adults had low ADL and IADL respectively. Various socio-economic, demographic, and health-related factors affect the ADL and IADL in older adults. Age and gender were found to be the most significant factors after other physical and mental health variables that are associated with the functional health in older adults, and the study showed that the likelihood of low ADL and IADL increases with increase in age. The prevalence and likelihood of ADL and IADL were found higher among women. The findings on the association of age and gender with low ADL and IADL corroborate with multiple past studies [31–37]. In contrast, a study by Zunzunegui et al. [38] reported that functional limitation decreased at every age, and improvement in ADL ability was observed among the elderly. A past study found that the age-standardized disability prevalence was highest for the rural female population [39]. The relative risk of the decline in functional ability with age was 2.0 for each 10-year increase in age [19].

In affirmation with the many previous studies, the present study also found various socio-economic factors like caste, marital status, economic status, place of residence, work status and educational attainment as other significant factors associated with the low ADL and low IADL scores [33, 34, 37, 40, 41]. With the increase in the educational attainment, the prevalence and odds of ADL and IADL limitation were also found to be decreasing, and similarly, it has been reported that poorly educated men have higher odds of functional limitations than well-educated men [41]. Our study noticed that working older adults had lower chances of having low ADL and IADL. The possible explanation could be that older adults were having pursuits of better ADL and IADL through employment participation that is one of the indicators of active ageing [42]. The study also showed that rural residents had 55% higher chances of having low ADL as compared to their urban counterparts, and the past literature also reveals that rural areas had higher rates of disability and urban males had shorter life expectancy with a disability as compared to rural males [39]. Factors like living arrangement and religion were not significant factors of low ADL and IADL in the present study when controlled for other socio-economic and health-related covariates. In contrast, previous studies have found the living arrangement of respondents as a significant factor, and the highest prevalence of disability was found among the elderly who lived alone [19, 43]. A previous study found that whereas factors like the living arrangement and self-rated memory had the highest impact on IADL, whereas pain, consuming more medications, and body mass index (BMI) had the highest impact on ADL disability [44].

The study also explored the impact of the number of pre-existing chronic conditions on the ADL and IADL disabilities in older adults and found it significant. The odds of having a low ADL was 1.156 times higher in those having more than three conditions compared to those with no pre-existing condition. A study by Bleijenberg et al. shows that respondents with more than or equal to 3 chronic conditions were 3 to 5 times at higher risk of developing disability as compared to those without any chronic condition [31]. The present study found that older adults with stokes and psychiatric diseases had the highest odds of low ADL and IADL. In line with the present study, a growing body of literature from different countries has demonstrated a strong association between the number of chronic conditions and poor self-rated health with poor physical functioning and disabilities including low ADL and IADL [19, 32, 45–48]. Previous studies have reported that chronic conditions related to musculoskeletal (arthritis, osteoarthritis, joint pain, etc.), cardiovascular disease, high depressive symptoms (anxiety, depression, etc.), hypertension, and even obesity have a strong association with higher ADL–IADL disability [14, 44, 46, 47, 49, 50]. It has been found that the risk of low ADL is related to duration and the time of the onset of chronic conditions as the risk was found to be more than twice as high in older adults who rapidly developed multimorbidity as compared to those who slowly accumulated diseases [10]. Multiple morbidities simultaneously affect various aspects of an individual’s health and act as a mediator between pathophysiological processes and negative health outcomes such as cognitive skills, functional limitations, and even results in a deteriorated quality of life [2, 10, 43].

The study found a significant gender difference in the prevalence and odds of ADL and IADL limitations where the female sex is at a disadvantage [17, 35, 51]. More females reported to have low ADL and IADL and were found to be 69% more likely to have low ADL as compared to males. After controlling for socio-economic and heath-related covariates and taking into account the number of chronic conditions (3+), it was found that males have higher odds of low ADL than females. On the other hand, IADL limitations were found higher among females. A recent study in affirmation reported that globally, the likelihood of having difficulties in IADL was about twofold higher for women than for men [40]. A past study, in contrast, explains that although there were differences in the socio-economic and health conditions between males and females, these conditions did not explain the gender difference in the prevalence of functional disability among Chinese adults [37].

It has been found in a past study that a higher risk of ADL disability in men aged 70–79 years is more associated with diseases like cancer, diabetes, and incontinence whereas, in women, pulmonary disease and diabetes are highly associated [35]. The possible reasons for these gender differences are explained by different studies in the past. It is delineated that the higher life expectancy along with the higher prevalence and severity of non-fatal disabling conditions like arthritis and musculoskeletal disease in older women are the leading reasons behind these gender differences in the functional limitations [43, 49, 51–53]. Gill et al. has found in their study that women were less likely to die due to disabilities and were more likely to experience moderate and severe disability trajectories as compared to men [52]. Also, it could possibly be due to the differences in the interrelated sedentary lifestyle, low physical activity and overweight [49, 53]. Other possible reasons that were found are that men tend to under-report their frailty, whereas women by nature are more likely to report or over-report ill health and disability [36, 40]. Generally, women have weaker physique, slower gait speed, weaker grip strength, and lower rates of recovery compared with men that influence the ADL and IADL limitations [45, 53].

This study has a few limitations as well. First, we were not able to establish a causal relationship between multimorbidity and the onset of ADL/IADL disability due to the cross-sectional nature of the data. However, our findings provide evidence on the contribution of most prevalent diseases, chronic multimorbidity, and different measures of functional limitation at the national level. Second, the self-reported information regarding chronic conditions and functional limitations may not be accurate and may instigate an information bias.

## Conclusion

The present study demonstrates a significant burden of functional limitations among older individuals and that there is a strong association between pre-existing chronic conditions and functional disability. This burden may increase in the near future and pose a serious public health threat to the economic development of the country if interventions are not formulated with target specific groups. Older adults with hypertension, diabetes, psychiatric disorders, heart disease, stroke, lung disease, or bone-related diseases should be effectively monitored to predict future functional limitations, which may lead to worsening health. Interventions are needed to orient health services for target-specific groups to prevent this avoidable cascade towards poorer health outcomes. Additionally, the results point out the other risk factors along with multiple diseases for functional limitations among older adults that can help in extensive and effective geriatric assessment and adopting multidimensional approaches.

## Supplementary Information

**Additional file 1: Table S1.** Logistic regression analyses of number of chronic conditions and functional limitations (AOR) among older adults.

## Data Availability

The study uses secondary data which is available on reasonable request through https://www.iipsindia.ac.in/content/lasi-wave-i.

## Abbreviations

AOR: Adjusted odds ratio
CI: Confidence interval
ADL: Activities of daily living
IADL: Instrumental activities of daily living
SRH: Self rated health
MPCE: Monthly per capita consumption expenditure
