# Supplementary material for: Number of chronic conditions and associated functional limitations among older adults: cross-sectional findings from the longitudinal aging study in India
Source: BMC Geriatr. 2021 Nov 23;21:664. doi: 10.1186/s12877-021-02620-0 (PMC8609791; doi:10.1186/s12877-021-02620-0)
Supplement: Supplementary file 1 — Additional file 1: Table S1. Logistic regression analyses of number of chronic conditions and functional limitations (AOR) among older adults. [file 12877_2021_2620_MOESM1_ESM.docx]

**Supplementary table**

| **Table-S1** Logistic regression analyses of number of chronic conditions and functional limitations (AOR) among older adults | | |
| --- | --- | --- |
| **Variables** | **Low ADL** | **Low IADL** |
|  | **AOR (95% CI)** | **AOR (95% CI)** |
| **Number of chronic diseases** |  |  |
| No disease | Ref. | Ref. |
| Single disease | 1.304*** (1.134 - 1.500) | 1.315*** (1.177 - 1.470) |
| Two | 1.744*** (1.481 - 2.053) | 1.513*** (1.331 - 1.721) |
| Three and above | 2.156*** (1.709 - 2.719) | 2.892*** (2.067 - 4.047) |
| **Age (in years)** |  |  |
| 60-69 | Ref. | Ref. |
| 70-79 | 1.407*** (1.225 - 1.616) | 1.446*** (1.274 - 1.641) |
| 80+ | 2.459*** (1.950 - 3.101) | 2.249*** (1.753 - 2.884) |
| **Sex** |  |  |
| Male | Ref. | Ref. |
| Female | 1.150* (0.997 - 1.327) | 1.631*** (1.454 - 1.829) |
| **Education** |  |  |
| No/Primary | Ref. | Ref. |
| Secondary | 0.733*** (0.615 - 0.874) | 0.622*** (0.526 - 0.736) |
| Higher | 0.637*** (0.489 - 0.831) | 0.437*** (0.353 - 0.542) |
| **Work status** |  |  |
| Never worked | Ref. | Ref. |
| Not working | 1.222** (1.045 - 1.428) | 1.222*** (1.057 - 1.413) |
| Working | 0.753*** (0.633 - 0.896) | 0.809*** (0.699 - 0.935) |
| Retired | 1.376** (1.071 - 1.767) | 1.001 (0.789 - 1.268) |
| **Marital status** |  |  |
| Currently in union | Ref. | Ref. |
| Not in union | 0.973 (0.832 - 1.137) | 1.260*** (1.097 - 1.447) |
| **Living arrangement** |  |  |
| Alone | Ref. | Ref. |
| With spouse | 1.072 (0.819 - 1.405) | 1.037 (0.809 - 1.331) |
| With others | 1.093 (0.873 - 1.368) | 1.016 (0.815 - 1.268) |
| **SRH** |  |  |
| Good | Ref. | Ref. |
| Poor | 2.030*** (1.797 - 2.293) | 1.710*** (1.519 - 1.923) |
| **Depression** |  |  |
| No | Ref. | Ref. |
| Yes | 1.933*** (1.618 - 2.309) | 1.730*** (1.472 - 2.034) |
| **Cognitive impairment** |  |  |
| No | Ref. | Ref. |
| Yes | 1.669*** (1.412 - 1.974) | 1.639*** (1.432 - 1.876) |
| **MPCE quintile** |  |  |
| Lowest | Ref. | Ref. |
| Lower | 0.955 (0.809 - 1.127) | 1.006 (0.883 - 1.147) |
| Middle | 1.025 (0.864 - 1.217) | 0.913 (0.789 - 1.058) |
| High | 0.901 (0.747 - 1.086) | 1.007 (0.845 - 1.198) |
| Highest | 0.904 (0.724 - 1.129) | 0.964 (0.805 - 1.155) |
| **Caste** |  |  |
| SC/ST | Ref. | Ref. |
| OBC | 0.910 (0.787 - 1.052) | 1.134** (1.011 - 1.273) |
| Others | 1.076 (0.924 - 1.252) | 0.985 (0.857 - 1.132) |
| **Religion** |  |  |
| Hindu | Ref. | Ref. |
| Muslim | 1.006 (0.856 - 1.184) | 0.950 (0.816 - 1.106) |
| Others | 0.882 (0.727 - 1.070) | 0.993 (0.843 - 1.169) |
| **Place of residence** |  |  |
| Rural | Ref. | Ref. |
| Urban | 1.116 (0.956 - 1.304) | 1.523*** (1.351 - 1.716) |
| *Ref: Reference; *if p<0.05, **if p<0.01, ***if p<0.001; AOR: Adjusted Odds Ratio; ADL: Activities of daily living; IADL: Instrumental activities of daily living; SRH: Self-Rated Health; MPCE: Monthly per capita consumption expenditure* | | |
